# Description of an establishment event by the invasive Asian longhorned beetle (*Anoplophora glabripennis*) in a suburban landscape in the northeastern United States

**DOI:** 10.1371/journal.pone.0181655

**Published:** 2017-07-20

**Authors:** Helen Hull-Sanders, Eugene Pepper, Kevin Davis, Robert Talbot Trotter

**Affiliations:** 1 United States Department of Agriculture, Center for Plant Health Science and Technology, Animal and Plant Health Inspection Service, Plant Protection and Quarantine, Otis Lab, United States of America; 2 United States Department of Agriculture, Animal and Plant Health Inspection Service, Asian Longhorned Beetle Cooperative Eradication Program, 151 West Boylston Dr., Worcester, United States of America; 3 United States Department of Agriculture Forest Service, Northern Research Station, Hamden, United States of America; Natural Resources Canada, CANADA

## Abstract

The establishment of non-native species is commonly described as occurring in three phases: arrival, establishment, and dispersal. Both arrival and dispersal by the Asian longhorned beetle (*Anoplophora glabripennis* Motschulsky), a xylophagous Cerambycid native to China and the Korean peninsula, has been documented for multiple locations in both North America and Europe, however the transitional phase, establishment, is not well understood for this species due to the need to rapidly remove populations to prevent dispersal and assist eradication, and the evident variation in the behavior of populations. Here we describe the dynamics of an establishment event for the Asian longhorned beetle in a small, isolated population within the regulated quarantine zone near Worcester, Massachusetts, USA. These data were collected during an opportunity afforded by logistical limits on the Cooperative Asian Longhorned Beetle Eradication Program administered by state, federal, and local government partners. Seventy-one infested red maple (*Acer rubrum*) trees and 456 interspersed un-infested trees were surveyed in an isolated, recently established population within a ~0.29 ha stand in a suburban wetland conservation area in which nearly 90% of the trees were host species, and nearly 80% were *Acer rubrum*. Tree-ring analyses show that within this establishing population, Asian longhorned beetles initially infested one or two *A*. *rubrum*, before moving through the stand to infest additional *A*. *rubrum* based not on distance or direction, but on tree size, with infestation biased towards trees with larger trunk diameters. Survey data from the larger landscape suggest this population may have generated long-distance dispersers (~1400 m), and that these dispersal events occurred before the originally infested host trees were fully exploited by the beetle. The distribution and intensity of damage documented in this population suggest dispersal here may have been spatially more rapid and diffuse than in other documented infestations. Dispersal at these larger spatial scales also implies that when beetles move beyond the closed canopy of the stand, the direction of dispersal may be linked to prevailing winds.

## Introduction

The movement of large quantities of commodities and people in the global economy has created opportunities for non-native species to transcend ecological barriers and be introduced to new environments. Many of these introductions do not lead to the establishment of new populations, and even when populations are established, many of these species may remain innocuous [1–2]. However, a minority of non-native species successfully colonize new habitats, and their presence leads to considerable ecological, economic and/or evolutionary impacts in the introduced range [2–5]. The Asian longhorned beetle, *Anoplophora glabripennis* (Coleoptera: Cerambycidae) is an example of one of these high-impact invaders. A polyphagous wood borer, Asian longhorned beetle larvae feed on a broad range of deciduous trees [6,7], resulting in extensive damage to both the cambium and xylem. Female feeding and oviposition has been documented on 43 species of trees [7–9] in 15 families, with larvae (the life stage responsible for much of the damage of concern) feeding most extensively on the preferred hosts *Acer* sp., *Populus* sp., *Salix* sp., and *Ulmus* sp. [10–13]. Within the beetle’s native range, which includes portions of China [14] and the Korean peninsula [11,12,15], outbreak populations of Asian longhorned beetles are most common in managed monocultures, windbreaks, and urban trees, which are often made up of non-native host species (frequently *Populus* sp.) [16], and the beetle is less common in stands of mixed native species.

Asian longhorned beetles are presumed to have been introduced to North American and European landscapes through international trade utilizing solid wood packing material [17–19] produced from infested wood. In many countries in Europe (including Austria (first reported 2001), Finland (2015), France (2003), Germany (2004), Italy (2007), Montenegro (2015), the Netherlands (2010), Switzerland (2011), and the United Kingdom (2011)), significant non-contiguous beetle infestations have been reported, and the potential for new detections continues. In North America, Asian longhorned beetles have been discovered in five states (Illinois (1998), Massachusetts (2008), New Jersey (2002), New York (1996), and Ohio (2011)) and Ontario, Canada (2003). North American infestations were first associated with cities with significant international trading centers, and their surrounding landscapes [20,21]. However, more recent infestations in Worcester, Massachusetts (2008) and Bethel, Ohio (2011) may be the result of the movement of international commodities beyond their port of entry to their consumer destination [20].

The process of invasion by non-native species can be classified into three stages: arrival (in which individuals are moved to regions outside of their native range), establishment (the process by which reproducing populations establish and stabilize at levels such that population decline and dissolution is unlikely), and spread (the range expansion of an invading species into novel areas) [5]. While arrival of Asian longhorned beetles can be inferred by the location of established populations, opportunities to understand the mechanisms driving establishment are more limited due to the high priority placed on the eradication of this species, which results in the rapid removal and destruction of infested trees following detection. This typically leaves little time to carry out intensive surveys and sampling to study infested landscapes in the early stages of establishment. As a result of these limits, past studies on beetle establishment and dispersal have either used mark-recapture methods in the beetles’ native range [22,23], statistical and graphical inference [24,25], or intensive reconstructions of beetle behavior based on damage within individual trees [26,27] in localized populations to identify patterns of beetle movement and dispersal.

Both the mark-recapture studies and the statistical inference models have provided new information regarding the distribution of risk on the landscape following the detection of the beetle. As is the case for many invasive species, prior to being identified as an environmental concern, basic ecological data describing beetle behavior and patterns of population dispersal were unavailable to guide Asian longhorned beetle eradication programs. In an effort to fill these knowledge gaps, Smith et al. [22] and Smith et al. [23] conducted large-scale mark-recapture studies on native beetle populations infesting non-native trees in Gansu Province, China. These studies found that 98% of the beetles that were recaptured were found within 920 m of the location where they were released, with a maximum observed dispersal distance of <2600 m. These early studies were critical in providing much needed information on dispersal; however, these studies were limited to assessing beetle behavior in native landscapes, and represented natural within-population dispersal rather than the patterns associated with range expansion. Due to the eradication status of the beetle in most introduced areas, mark-recapture studies are not an option as live beetles cannot be released in areas under eradication.

Statistical and graphical methods are applicable in invaded landscapes, and can often take advantage of data collected by eradication programs [24,25]. However, these studies often require extensive datasets, and the models may provide an assessment of beetle movements at large spatial scales. When isolated pockets of infestation are found, survey and eradication efforts are typically focused at the stand-scale. Filling this knowledge gap requires reconstructions of beetle population development within and between trees.

Two studies have sought to fill this stand-scale knowledge gap [26,27]. These studies have taken advantage dendrochronological methods to date the damage caused by ovipositing females and emerging adults within a stand. The work by Turgeon et al. [26] provided a reconstruction of beetle population growth and movement in an isolated infestation near Toronto, Canada, and revealed the infestation had been in place for 10 years during which the beetles had spread to infest 50 trees. However, within the infestation, the vast majority (more than 800) of the adult beetles had emerged from a single tree, while 1 tree had more than 30 exit holes, 2 trees had 10–30 exit holes, and 10 trees had fewer than 10 exit holes. This concentration of exit holes on a focal tree suggests the population had been slow to spread, and that many of the adult beetles remained on their natal host. A second reconstruction of beetle establishment and spread was carried out at Paddock Wood in the United Kingdom by Straw et al. [27]. This study found similar patterns; a 10-year old population which had spread to include 66 infested trees. Like the population in Canada, the beetles had also remained primarily on a focal tree from which the majority of the adult beetles (nearly 500) had emerged. Only one additional tree in the stand had more than 10 exit holes. These studies suggest several common patterns including a relatively slow spread of infestation of new trees and a tendency for the adults to remain on their natal tree.

While these studies have expanded our understanding of the local dynamics of Asian longhorned beetle population growth and movement, both of these studies represent single sample points, and patterns may be highly dependent on local conditions. Here, we seek to expand our understanding of beetle behavior and assess some of the general patterns previously observed by assessing population establishment and movement in an isolated pocket of infested trees identified by the United States Department of Agriculture (USDA) Asian Longhorn Beetle Eradication program in 2013 near Worcester, Massachusetts, USA. The identification of this isolated infestation, and logistical delays in its removal provided a brief window of time in which the site could be surveyed in detail before the removal and destruction of infested material. Within the isolated plot, all host and non-host trees were surveyed and beetle damage was dated using basic tree ring methods [28] to reconstruct the pattern of establishment within that stand. The identification of a nearby, isolated pocket of infested trees also provided an opportunity to evaluate landscape factors such as wind that may drive larger-scale patterns of beetle dispersal. By generating a spatial analysis of host tree usage, the information on early within-stand beetle movement enables managers to assess the potential for beetle dispersal in a novel landscape, and in turn provides additional biological information on which to base risk assessments and management strategies.

## Methods

### Data sources

Infested trees were identified by surveys carried out by the Massachusetts division of the Asian Longhorned Beetle Eradication Program. This program, which is jointly managed by the USDA Animal and Plant Health Inspection Service (APHIS) and the Massachusetts Department of Conservation and Recreation (Mass DCR), conducts surveys using ground-based teams using binoculars and aerial teams of tree climbers to identify infested trees for removal and disposal. When infested trees are found, personnel with the eradication program assign each tree a unique program identification number and document the tree’s genus and, (when possible) species, as well as the diameter at breast height (dbh), and location of the trees using handheld GPS units.

### Site description

**Fig 1:**
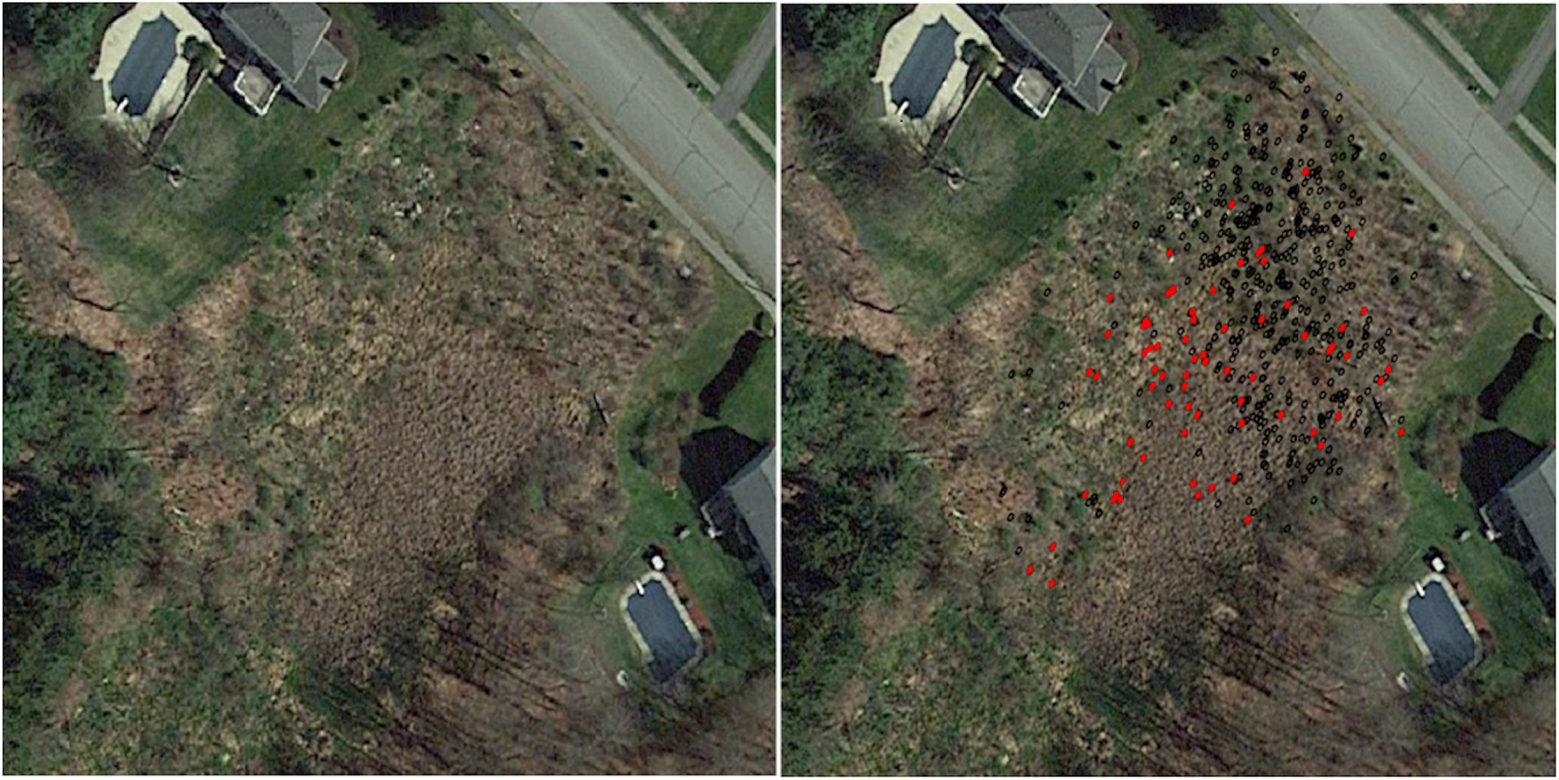
Aerial imagery of Shrewsbury, MA (from Image MassGIS, www.mass.gov). A) After tree removal April 2014 and B) before tree removal August 2013 with red circles indicating the location of Asian longhorned beetle infested trees.

The stand of infested trees surveyed for this analysis occupied an overall area of ~ 2,860 m^2^ in a wetland conservation area (Fig 1) owned by the Town of Shrewsbury, Massachusetts, U.S.A. (Town of Shrewsbury, Taxplate 29, Block 05100). Work performed under Massachusetts general law chapter 132 section 8 and 11. Inspectors with the Massachusetts Asian Longhorned Beetle Eradication Program found the study stand imbedded within a suburban environment in Shrewsbury, MA, within the Asian longhorned beetle regulated area. The current regulated area includes ~ 285 km^2^ of urban, suburban, and wooded landscapes centered on Worcester, MA, an urban environment which grades rapidly into the surrounding forested landscape. The first infested tree to be detected in the Worcester, MA area was found in early in the winter of 2008 and since then, ~25,000 infested trees have been identified and (along with ~11,000 trees considered to be at high risk for infestation) destroyed by the eradication program [29]. Among the documented infested trees, nearly 97% have been in the genus *Acer*.

### Stem-mapping

The stand described above included 526 individual tree stems with inter-tree distances ranging from 0.1 m– 18.5 m. While the canopy of most of the stand was closed and stem density was high, the understory was open and relatively unobstructed, with visibility to ~ 70 meters. The effect of wind was evident in the upper canopy as indicated by the movement of limbs and leaves, however, within the stand, little air movement was noted.

The dense, closed canopy, and close proximity of the stems made the mapping of the stem locations by GPS unfeasible, so locations were mapped using a standard bearing and distance method [30]. The method consisted of establishing 1 primary control point, and 4 sub-control points. The distance and bearing for each of the sub-points from the control point was determined using a level transit (Topcon AR-F6 Auto Level). The distance and bearing from one of these points was then determined for each stem, using the same equipment. Bearings were taken to the center of the stem to the nearest 1/10^th^ of a degree, and distances were determined to the nearest 0.1 m. From these data, the locations were converted to Cartesian coordinates relative to the primary control point. These Cartesian coordinates were then converted to UTM coordinates using a single GPS coordinate collected at the primary control point. The primary control point location was determined using a Trimble Geo Explorer 3000 Series operating with Windows Mobile 6 and ArcPad ver. 10 [31]. The coordinate was determined in UTM Zone-19, and was estimated by spatially averaging 1,000 points (maximum Positional Dilution of Precision allowed was 7). Converted tree coordinates were then used to create a shapefile using ArcMap V10.2 [32], and overlaid on aerial photographs to produce Fig 1. Measurements for tree diameters at breast height (dbh) were recorded for all trees; comparisons between distributions of infested and un-infested tree dbh values were made using the Wilcoxon signed rank statistic using R statistical package [33].

### Identification of infested trees

Two distinctive indicators of beetle presence were used to diagnose infestation. Initially, female beetles will chew an elliptical oviposition pit through the bark into which she will typically oviposit a single egg. After development within the tree, adult Asian longhorned beetles will emerge, creating a nearly perfectly round exit hole ~1 cm diameter. Aerial inspectors climbed, surveyed, and verified all infested and non-infested trees within 25 m radius from any tree with an exit hole. Tree sections with oviposition pits and exit holes were isolated after the tree was felled and placed into quarantine for determination of aging characteristics (*see* Dating Beetle Damage below).

### Beetle infestation levels

The severity of the infestation within individual trees was defined by identifying the type and abundance of beetle damage. Trees which have oviposition pits but no emergence holes were identified with infestation level of A, trees with 1–10 emergence holes and trees with 11–100 emergence holes were identified as B and C level infestations, respectively. Although not explicitly documented, the locations of the observed exit holes and oviposition pits were limited to the main stem and lower canopies of trees. Exit holes and oviposition pits were not found in the upper canopy.

### Wind analysis

Daily records for the direction of the maximum 5-s wind for the assumed beetle flight seasons (June through October, inclusive) from 1998 through 2014 (estimated duration of infestation in Worcester, MA regulated area) were obtained from the National Oceanic and Atmospheric Administration (NOAA) National Climatic Data Center (NCDC, available at https://www.ncdc.noaa.gov), these records provide the directionality of daily wind gusts. Data used for analysis were obtained from the NOAA station at the Worcester Regional Airport (Station No.: GHCND: USW00094746) located to the southwest of the center of the Asian longhorned beetle infestation in Worcester County, MA, and ~14.5 km west-southwest of the study stand. This is the closest meteorological station with complete records within the Asian longhorned beetle regulated area. Daily values for the full record set (records were complete) were tabulated to provide a probability distribution for wind directionality.

**Fig 2:**
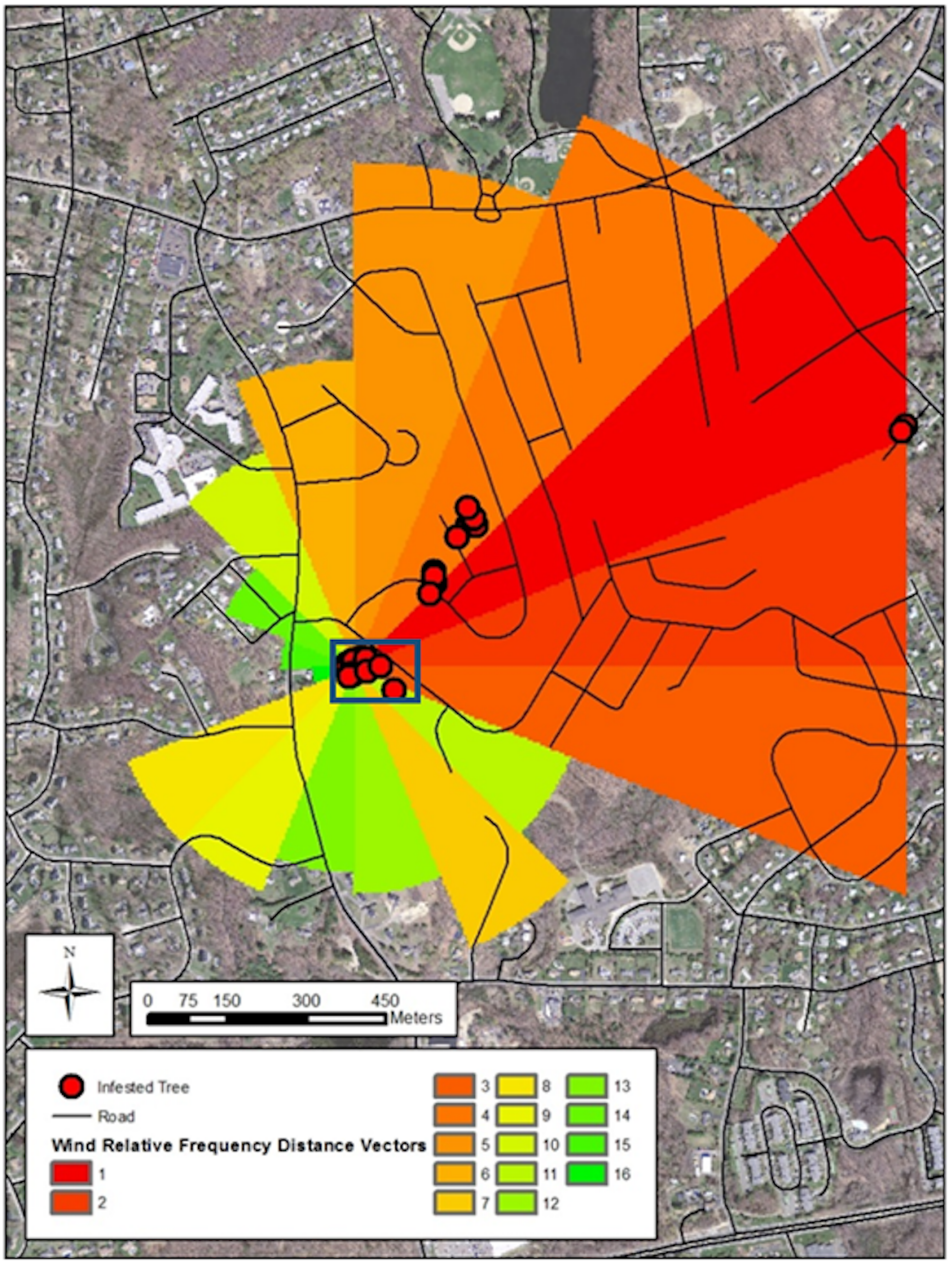
Wind direction frequency analysis. Wind relative frequency distnace vectors overlaid onto a map of the infested area, Shrewsbury, MA USA. Red circles indicate location of Asian longhorned beetle infested trees, the callout box indicates the area in Fig 1.

Wind source frequency was calculated based on 22.5° bins to generate a “wind” direction bins. Destination Frequency (the direction the beetle would be expected to disperse) was calculated as the inverse of the Wind Source Frequency (Fig 2). The 16 direction bins were then ranked by frequency (with two bins tied for rank 10). Dominant wind direction was plotted in ArcGIS Ver. 10.2 [32].

### Dating beetle damage

Asian longhorned beetle damage was dated using standard dendrochronological techniques as described in Turgeon et al. [26] and Straw et al. [27]. Sections of trees with visible exit holes, both healed and unhealed, were removed from all B and C level trees in the field October 2013. Trees were also sampled to identify the oldest oviposition pits (based on weathering and wound response). Sections were placed in a kiln the same day they were removed at the USDA APHIS Plant Protection and Quarantine (PPQ) quarantine facility, Barnstable County, Massachusetts. The kiln was held at 80° C for 15 h to ensure all beetles had been destroyed. The wood was then allowed to continue to dry at room temperature for an additional 72 h. Using a band saw, wood sections were cross-cut through the Asian longhorned beetle exit holes or oviposition pits, and the surfaces were sanded to reveal growth rings. Rings beyond the edge of the emergence holes were counted to determine the year of beetle emergence. Holes with no evidence of healing were dated as the current year. Rings formed over healed or partially healed exit holes were counted to determine the year of emergence.

### Establishment distances

A heavily infested tree (42 visible exit holes and 30 oviposition pits) assumed to be the source of the subsequent infestation was confirmed by dendrochronological methods to be the earliest infested tree (2008 oviposition, 2010 exit). The Kolmogorov-Smirnov test for distribution differences was used to determine if beetles moved randomly throughout the stand using R statistical package [33]. Expected dispersal distances were calculated using the source tree and finding the distance to all other trees in the stand, both infested and un-infested. Observed dispersal distances were calculated by determining distances between the source tree and the infested trees only.

### Adjacency rules

The movement of female beetles from the natal tree to a newly infested tree is used here to define connections, or adjacencies among trees. The movement of beetles is inferred based on two rules established by Trotter and Hull-Sanders [24], those rules are: 1) host trees receive beetles from the nearest tree with a higher level of infestation (which is assumed to be older) and 2) trees can serve as the source of beetles for multiple trees, but each tree is assumed to have received beetles once.

The lengths and directions of these inferred dispersal events (adjacency vectors), and the dispersal distance percentiles were calculated using a custom built MatLab R2013b [34] script (TreeAdjacency) as described in Trotter and Hull-Sanders [24]. Distance vectors were tabulated to produce models relating vector distance to frequency.

## Results

The infested stand in Shrewsbury, MA included 526 trees within a ~ 2,860 m^2^ area (Fig 1), and included trees in the genera *Acer*, *Betula*, *Carya*, *Cornus*, *Fagus*, *Fraxinus*, *Ilex*, *Malus*, *Pinus*, *Populus*, *Quercus*, *Salix*, *Ulmus*, and *Viburnum*. Host genera (as listed by Meng et al. 2015), including *Acer*, *Betula*, *Fagus*, *Fraxinus*, *Malus*, *Populus*, *Salix*, and *Ulmus* accounted for 94% of the stems. Red maple (*A*. *rubrum)* alone accounted for 81% of the stems while *Malus* spp. accounted for 8%. *Acer platanoides*, *Populus tremuloides*, and *Salix* spp. were represented by a single stem each. The earliest exit hole dated to 2010 and corresponded with the earliest oviposition pit dated to 2008, suggesting a ~2 year period for development. A total of 71 trees had signs of infestation (oviposition pits and/or exit holes) at the Shrewsbury location, all of these were *A*. *rubrum*. In total, the stand included 2 trees with 42 and 40 exit holes, 4 trees with 2–4 exit holes, and 65 trees with oviposition pits only. Within the Asian Longhorned Beetle Eradication Program, these are referred to as C, B, and A level infestations, respectively. The remaining host trees did not show indications of infestation.

**Fig 3:**
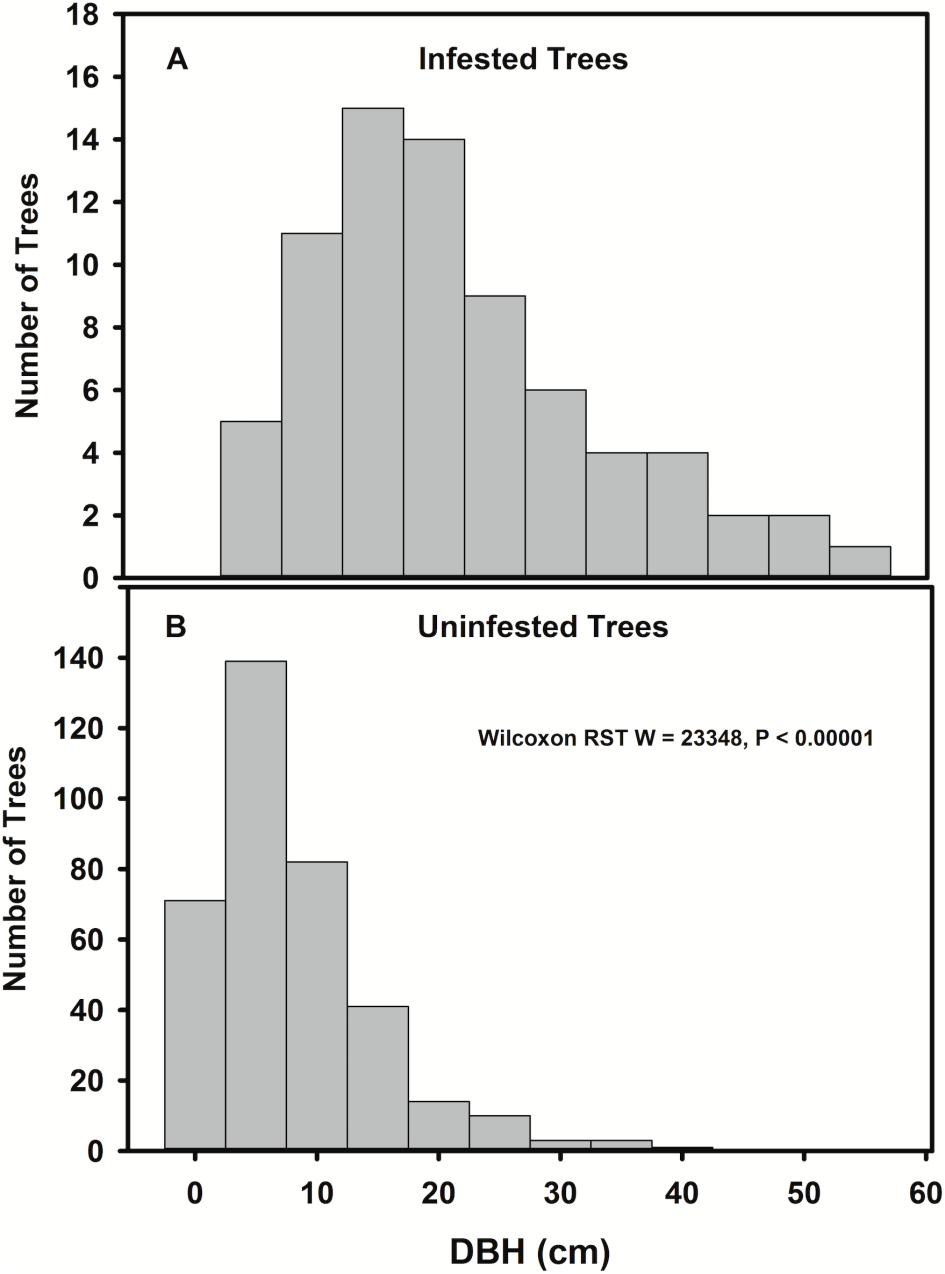
Distribution of Asian longhorned beetle. Infested (A) and un-infested (B) trees based on the diameter at breast height (dbh) in centimeters for 527 trees in Shrewsbury, MA USA.

**Fig 4:**
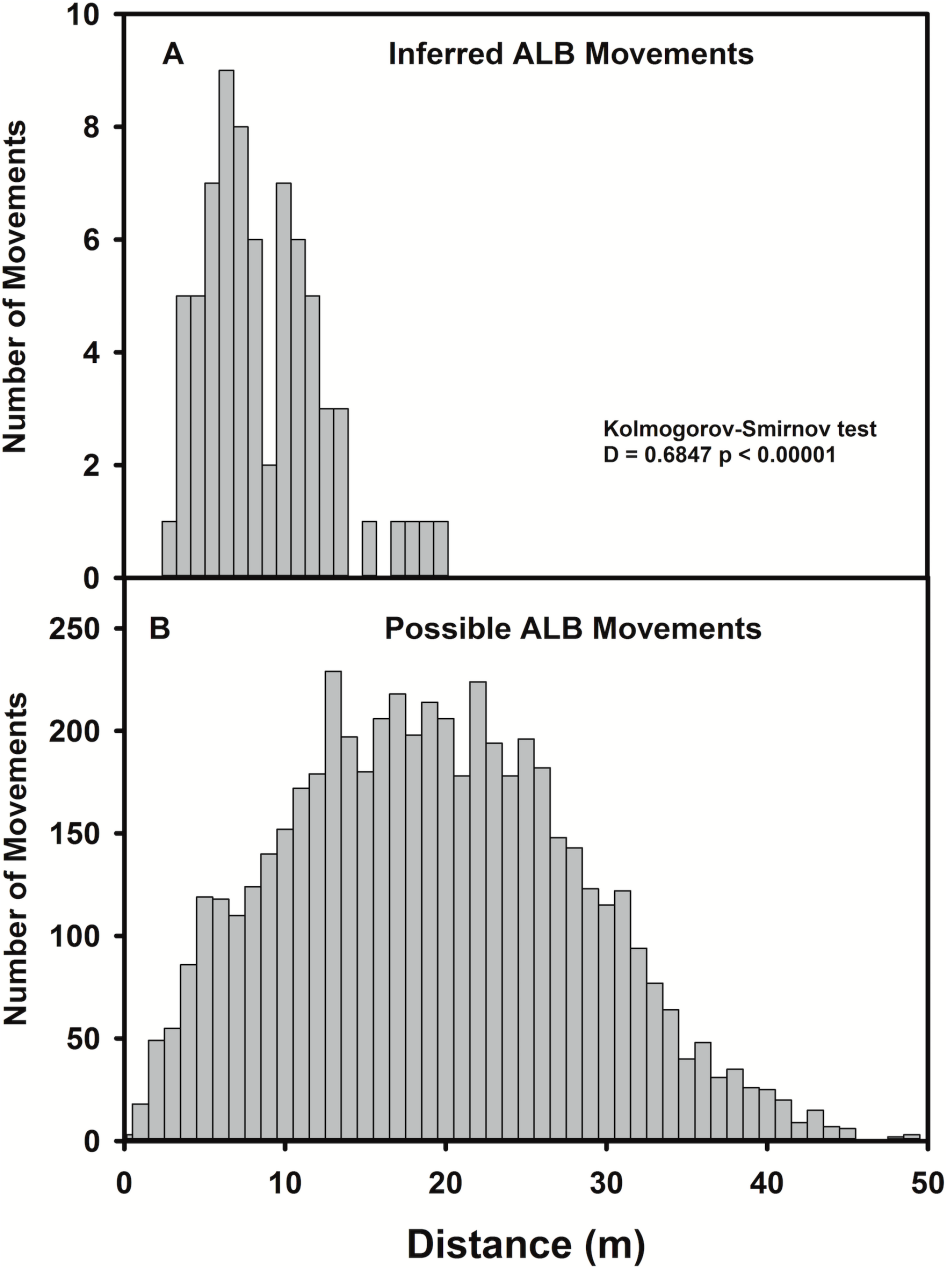
Dispersal movement of Asian longhorned beetle. Inferred (A) and Potential (B) below canopy dispersal movements away from source trees (>10 exit holes) to surrounding host trees.

**Fig 5:**
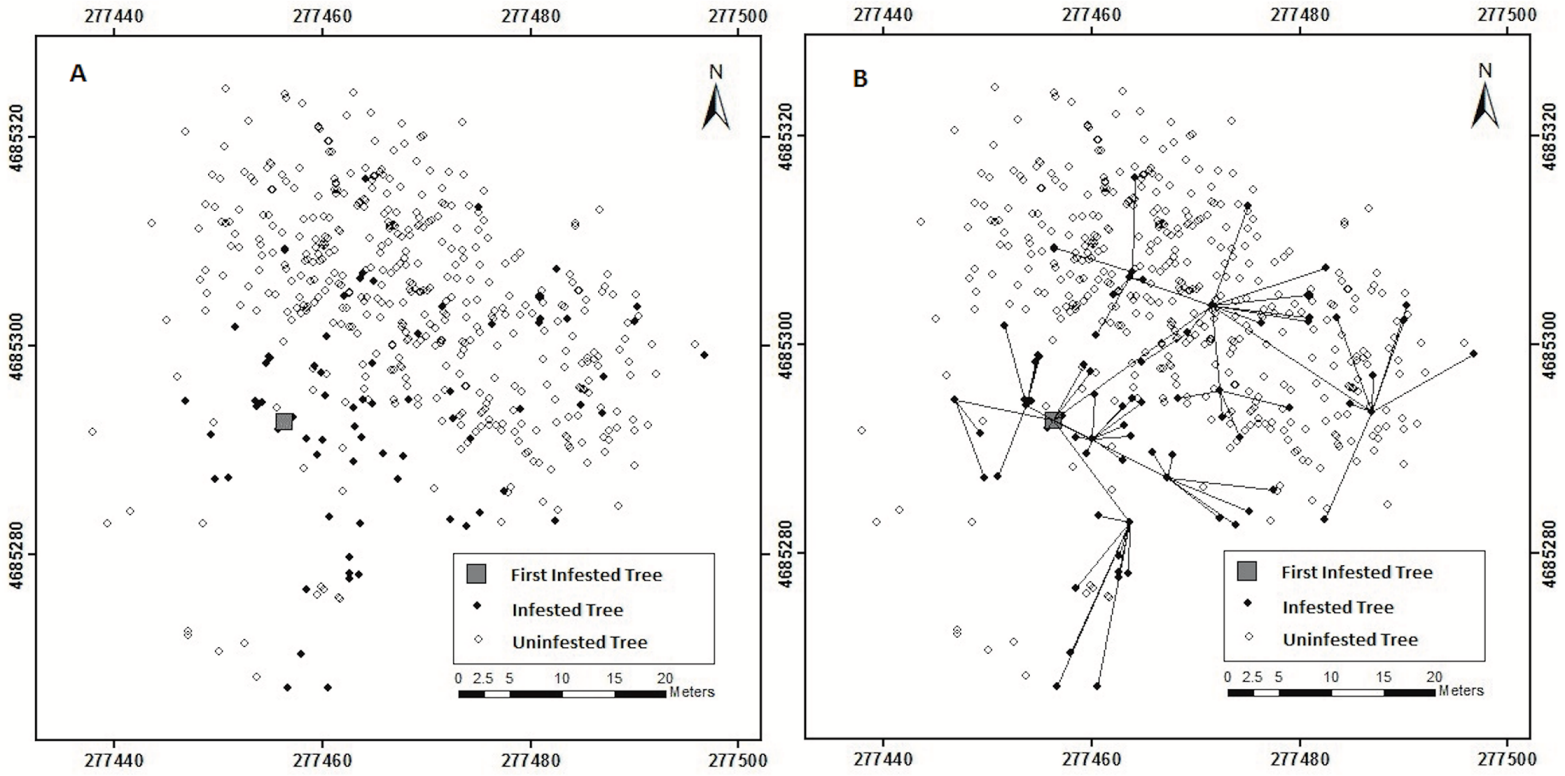
Relative location of trees and Adjacency depicting inferred dispersal. Within the stand, open circles represent un-infested trees and filled circles represent infested trees. (A) Large square represents the assumed source for the infestation (initially infested tree based on exit hole data). (B) Lines connecting trees indicate inferred patterns of Asian longhorned beetle movement through the stand.

Infested *A*. *rubrum* host trees were, as a group, larger (median = 19.5 cm dbh, IQR = 15.2 cm dbh) than un-infested *A*. *rubrum* (median = 6.0 cm dbh, IQR = 8 cm dbh) (Wilcoxon rank sum W = 23348, *P* < 0.0001) (Fig 3). The distribution of distances that beetles were inferred to have moved differed significantly from the distances that might be expected if movements were to occur at random (Kolmogorov-Smirnov D = 0.6847, *P* < 0.0001) (Fig 4), with beetles moving longer distances than might be expected if beetles were to disperse randomly through the stand (median inferred movement distance 5.74 m, IQR = 5.84 m, median possible inter-tree movement distance 18.89 m, IQR = 13.13 m). Movement among trees by adult females was inferred by using assumptions described in Trotter and Hull-Sanders [24], in which beetles on A trees are assumed to have originated from the nearest B or C tree, and beetles on B trees are assumed to have originated on the nearest C tree. These assumptions of tree adjacency (connectivity) are shown graphically on the Fig 5B by the lines, which denote the movement of beetles to B and A trees.

Wind patterns on the landscape during the emergence months were generally SSW to NNE (Table 1; Fig 2); this probability distribution provides an expected distribution of dispersal directions from a given point source (Table 1). When the resultant polygon was applied at the county level to the initially infested tree within the study stand, more distal infested trees identified by the eradication program generally fell within four of the most common wind direction bins, between absolute bearings 45° and 112.5° (Fig 2).

**Table 1:** Wind source and destination obtained from the NOAA station at the Worcester Regional Airport (Station No.: GHCND: USW00094746) for 1998 through 2014.

	Wind Source	DEGREES (From North)	Wind Direction (Destination)	Wind Source Frequency	Percentage	Rank
1	N	0–22.5	S	110	4.13%	5
2	NNE	22.5–45	SSW	99	3.71%	4
3	NE	45–67.5	SW	117	4.39%	8
4	ENE	67.5–90	WSW	118	4.43%	9
5	E	90–112.5	W	20	0.75%	1
6	ESE	112.5–135	WNW	36	1.35%	2
7	SE	135–157.5	NW	68	2.55%	3
8	SSE	157.5–180	NNW	113	4.24%	7
9	S	180–202.5	N	149	5.59%	11
10	SSW	202.5–225	NNE	245	9.19%	12
11	SW	225–247.5	NE	289	10.84%	13
12	WSW	247.5–270	ENE	372	13.96%	16
13	W	270–292.5	E	351	12.17%	15
14	WNW	292.5–315	ESE	319	11.97%	14
15	NW	315–337.5	SE	113	4.24%	6
16	NNW	337.5–360	SSE	146	5.48%	10

Frequencies were determined by counting the number of fastest 5- second wind intervals. Sixteen directional bins ranked by wind frequency ranked the lowest (1) to highest (16) wind destination likelihood.

At smaller spatial scales, if the establishment of new pockets of infestation are the result of stratified dispersal from the source, then it would be expected that these pockets of infestation would be younger than the source population, as indicated by the number of infested trees and exit holes. Similarly, if adult dispersal is influenced by wind direction then new infestations may be expected to be associated with wind patterns. An assessment of the survey records for the Asian Longhorned Beetle Eradication Program shows that two trees which were ~1400 m from the surveyed population were found to be infested, with both trees having only had small numbers of oviposition pits. Trees closer to the infestation, but outside the surveyed stand had more severe damage, including a greater number of oviposition pits, feeding galleries, sapwood entrance holes, and a few exit holes. All infested trees were located within the top 4 wind destination categories, including the two outliers shown in Fig 2, suggesting outside of the stand, wind direction may play a role in structuring dispersal.

## Discussion

Past research has shown the dispersal behavior of Cerambycid beetles to be variable, often dispersing when host quality goes down, adult population densities increase, or inter-specific competition with other large cerambycids occurs [35–39]. Recent studies by Turgeon et al. [26] and Straw et al. [27] have provided reconstructions of the spread of the Asian longhorned beetle within infested stands, and these data have also suggested beetles may remain on the natal tree, leading to high beetle density in focal trees within an isolated infestation. However, the data from the Worcester, MA stand seems to indicate that this may not be a rule in Asian longhorned beetle population dynamics. Here, the beetles seemed to move rapidly to infest numerous trees within the stand, and perhaps some trees well beyond the stand, long before populations in the originally infested tree grew to the sizes observed in Canada or the United Kingdom. Weather, stand structure, tree density, and the nature of the landscape may play a role in structuring these patterns. Although each of these three infestations was found in urban environments, the structures of the landscapes varied. The infestation in Canada was found in an area described as “light industrial” [26], while the infestation in the United Kingdom was found in an area with “light industry” as well as agricultural lands and woodlots [27]. The localized infestation described in this study was found in a heavily wooded residential area, which lacked industry. In all of these locations, there exists the potential for beetles to disperse under their own power; to be moved by transport of infested materials or by hitch-hiking on vehicles; however, limited replication (3 locations) and lack of direct observations mean that the role these variations in the landscape play in the active and passive dispersal of the beetle remain unknown, but clarifying this pattern may be important in providing guidance to management and eradication programs, as the propensity of the beetle to disperse is a key issue in determining the needed extent and intensity for effective surveys.

The study by Trotter and Hull-Sanders [24] highlights the potential importance of this information. At the time the study was conducted, limited information on the propensity of adult female Asian longhorned beetles to dispersal from natal trees in the introduced range was available. To accommodate this knowledge gap, the potential patterns of beetle distribution were inferred based on two sets of assumptions. Under the strict assumptions of dispersal, it was assumed that the beetle would be reluctant to disperse from trees with low levels of infestation, a pattern consistent with that observed by both Turgeon et al. [26] and Straw et al. [27]. This set of assumptions, by the nature of the distribution of the infested trees, means that while fewer beetles disperse, the dispersal events that do occur are longer. Under the alternative, relaxed dispersal scenario, it is assumed that beetles may disperse from trees with low levels of infestation. This pattern results in more trees acting as sources for dispersing beetles, but implies that when beetles do disperse, they typically do so over shorter distances. This relaxed pattern of dispersal assumptions is more in alignment with the patterns observed within this study. Ultimately, these patterns may alter the spatial distribution of beetle risk on the landscape around infested areas.

Other studies conducted in the beetles’ native range may shed some light on this issue. In the introduced range, under strict dispersal rules (i.e. beetles do not disperse from the tree from which they emerged until the tree has been heavily infested) dispersal distances were estimated to be greater than 2.3 km based on infested tree data [24]. In trees within an agricultural wind-break in China, Smith et al. [22] observed 98% of marked Asian longhorned beetles dispersed within 920 m of their release point; however, a minority could be found as far as 2.6 km [23] and the direction of dispersal was associated with wind direction [40]. The outlying population of recently infested trees (oviposition pits only, no exit holes), which are aligned with the direction of prevailing daytime winds supports both the direction of movement, and the indication of long-distance dispersal events. The association with wind direction suggests long-range dispersers may have left the stand, perhaps by flying above the canopy where they would have been subjected to stronger winds. Although it is possible that the ENE infestation of two trees >1400 m from the surveyed area was due to human mediated movement, the two trees lie within the dominant wind vectors and are within dispersal distances inferred by Trotter and Hull-Sanders [24], suggesting spread may have been driven by natural dispersal.

The limited distribution of infested trees observed in both Canada and the UK do not necessarily suggest the absence of long distance dispersal. In fact, Turgeon et al. [26] discuss the likelihood that the isolated infestation studied was the result of long-distance dispersal by beetles from the center of a larger infestation in Toronto, Canada, some 10 km away. This process, by which larger infestations produce satellite infestations is also likely to have produced the pocket of Asian longhorned beetle described in this study. What is not known however, is why these two satellite infestations, with similar climatic conditions, seem to have resulted in different patterns of beetle movement.

While most non-indigenous species fail to establish after arrival, problematic alien species become invasive after both establishing and dispersing following introduction [5]. Short-distance dispersal may be part of the establishment phase. The establishment phase serves as the decisive interval during which expanding populations stabilize and increase their distribution such that population decline and dissolution is unlikely [5]. Unfortunately, management and subsequent eradication programs often only detect the problem during the spread phase and have little opportunity to determine, control, or understand the establishment phase. Our survey of all standing trees within the conservation area before the Asian longhorned beetle population was expected to substantially increase (within the first five years after arrival [41]) allowed us to characterize the establishment phase.

Three major patterns of Asian longhorned beetle movement following establishment within a stand have emerged. The first, is that the pattern of strict beetle movement, in which dispersal from the natal tree is unlikely until the tree has been heavily utilized has been supported by some studies, however, the current study suggests this pattern may not be consistent among all locations. The second issue of interest is that, once a beetle does disperse from its natal tree, it does not appear to randomly select a new host, but may be selective based on characteristics such as size and species, with an emphasis on *A*. *rubrum*. This finding agrees with previous studies such as Dodds et al. [42], which found Asian longhorned beetles in mixed stands with *A*. *rubrum*, *Acer saccharum*, and *Acer platanoides*, tended to infest *A*. *rubrum* trees with diameters similar to infested trees found at our site (mean dbh Delaval 17.0 ± 1.2 cm, Boylston 19.2 ± 2.3 cm, this study 21.8 ± 1.3 cm). Asian longhorned beetles may bypass smaller host trees that may be located closer in proximity to the natal tree, continuing to move through the understory until “appropriate” hosts are found. If this behavior is driven by the suitability of the host for the development of the larvae, this pattern would suggest a potential preference-performance link [43], though more research on this is needed. While female Asian longhorned beetles will create significantly more oviposition pits and deposit more viable eggs on *A*. *saccharum* in the lab (Smith et al. 2002) [44] and in the field [45] than on *A*. *rubrum*; in the field, similar sized *A*. *rubrum* trees had a greater number of exit holes [45]. Dodds and Orwig also [45] found that within two infested stands in Worcester, MA, only *Acer* tree species were infested. All of the trees at one site and more than half of the trees at a second site >30 cm dbh were infested. However, these infestations were larger and presumably older than the infestation described in this paper. The potential interaction between host size and host species in determining host quality may help structure the dynamics of invasions where the beetle and hosts may lack an evolutionary history such as in the invaded stands in Canada, the United States, and throughout Europe, and merits further study.

These results may have application to the management of Asian longhorned beetles, and provide additional information on how beetle populations may behave when establishing in novel environments. However, these data suggest inconsistencies among invaded stands, and highlight the potential for as-yet undocumented factors to play a large role in how beetle move through the landscape. This information in turn is likely to be of value for basic biogeographic theory, as it may help explain variations in dispersal patterns, and some of the factors (and evolutionary history, such as host associations) which may drive that variation. This information is also likely to be critical for management and eradication efforts, where the effective use of surveys depends on a sound understanding of the probabilities associated with the directions, distances, and frequencies of propagule movement, and survey efforts may find new efficiencies from increased focus on tree size and local wind patterns in identifying the boundaries of infestations, though additional work at multiple spatial scales is needed to parameterize survey priorities.
